# Microglia processes associate with diffusely injured axons following mild traumatic brain injury in the micro pig

**DOI:** 10.1186/s12974-015-0405-6

**Published:** 2015-10-06

**Authors:** Audrey D. Lafrenaye, Masaki Todani, Susan A. Walker, John T. Povlishock

**Affiliations:** Department of Anatomy and Neurobiology, Virginia Commonwealth University Medical Center, P.O. Box 980709, Richmond, VA 23298 USA; Advanced Medical Emergency and Critical Care Center, Yamaguchi University Hospital, Yamaguchi, Japan

**Keywords:** Mild traumatic brain injury, Diffuse axonal injury, Neuroinflammation, Microglia, Micro pig

## Abstract

**Background:**

Mild traumatic brain injury (mTBI) is an all too common occurrence that exacts significant personal and societal costs. The pathophysiology of mTBI is complex, with reports routinely correlating diffuse axonal injury (DAI) with prolonged morbidity. Progressive chronic neuroinflammation has also recently been correlated to morbidity, however, the potential association between neuroinflammatory microglia and DAI is not well understood. The majority of studies exploring neuroinflammatory responses to TBI have focused on more chronic phases of injury involving phagocytosis associated with Wallerian change. Little, however, is known regarding the neuroinflammatory response seen acutely following diffuse mTBI and its potential relationship to early DAI. Additionally, while inflammation is drastically different in rodents compared to humans, pigs and humans share very similar inflammatory profiles and responses.

**Methods:**

In the current study, we employed a modified central fluid percussion model in micro pigs. Using this model of diffuse mTBI, paired with various immunohistological endpoints, we assessed the potential association between acute thalamic DAI and neuroinflammation 6 h following injury.

**Results:**

Injured micro pigs displayed substantial axonal damage reflected in the presence of APP+ proximal axonal swellings, which were particularly prominent in the thalamus. In companion, the same thalamic sites displayed extensive neuroinflammation, which was observed using Iba-1 immunohistochemistry. The physical relationship between microglia and DAI, assessed via confocal 3D analysis, revealed a dramatic increase in the number of Iba-1+ microglial processes that contacted APP+ proximal axonal swellings compared to uninjured myelinated thalamic axons in sham animals.

**Conclusions:**

In aggregate, these studies reveal acute microglial process convergence on proximal axonal swellings undergoing DAI, an interaction not previously recognized in the literature. These findings transform our understanding of acute neuroinflammation following mTBI and may suggest its potential as a diagnostic and/or a therapeutic target.

**Electronic supplementary material:**

The online version of this article (doi:10.1186/s12974-015-0405-6) contains supplementary material, which is available to authorized users.

## Background

Mild traumatic brain injury (mTBI) is a common insult that exacts devastating personal and social costs [1–4]. Diffuse mTBI is typically caused by acceleration-deceleration forces, such as those encountered during a motor vehicle accident or sports-related event. The forces generated by the rapid movement of the brain within the cranial vault lead to a multitude of complex metabolic, physiologic, and pathologic responses [5–9]. One pathology that frequently follows mTBI is diffuse axonal injury (DAI), in which force-induced stress results in discrete areas of scattered axonal disruption that ultimately progress to disconnection. This results in a proximal axonal segment that remains connected to the neuronal soma and a distal segment that progresses to Wallerian degeneration [10–13]. Advanced neuroimaging and histological studies have firmly established a positive correlation between DAI and TBI-induced morbidity both clinically and experimentally [10, 14–18].

In addition to DAI, progressive chronic neuroinflammation has been observed following TBI with the suggestion that this pathology is also associated to morbidity [19–24]. Neuroinflammation involves the activation of resident brain microglia and later includes systemic infiltrating macrophages in injuries that involve blood–brain barrier disruption. Upon activation/reactivation, resident microglia undergo morphological changes associated with activation, transforming from highly ramified “resting” or “surveying” phenotypes to activated microglia, with truncated processes, larger cell bodies, and less complex process networks and/or amoeboid morphologies [25–27]. Activated microglia are associated with a variety of detrimental as well as regenerative functions, including phagocytosis, cytokine secretion, and/or neurotrophin secretion [28–34]. To date, the majority of studies exploring neuroinflammatory responses to TBI, including our own, have focused on the phagocytic role of microglia in clearing Wallerian debris days following injury [29, 35–39]. Knowledge is, however, greatly lacking regarding any potential association between microglia and the proximal segment of axons undergoing DAI, independent of the degenerating distal axonal segment and/or Wallerian change.

Previously, our lab reported that while highly activated phagocytic microglia were commonly seen associated with distal degenerating axonal segments, little association was found between activated microglia and proximal axonal segments undergoing DAI [36, 39]. These studies, however, were not designed to address the aforementioned issue in that our previous studies were confined primarily to sub-acute and chronic post-injury time points. Thus, these studies provided no information regarding more acute neuroinflammatory changes or any potential relation to early DAI. Further, while our previous study utilizing transgenic mice allowed for the precise discrimination between proximal and distal axonal segments undergoing DAI, analysis was restricted to the optic nerve [36], a unique white matter region containing a homogeneous axonal population with potentially distinctive microglial responses to injury [40–42]. Lastly, as with the majority of published work in this area, our previous studies explored TBI-related neuroinflammation in rodents, whose systemic inflammatory responses are known to differ from humans [43–46]. In consideration of the fact that pig inflammatory profiles are much more human-like [47, 48], we revisited the issue of microglial response to DAI acutely following injury in a micro pig model of mTBI.

Using an adapted central fluid percussion injury (cFPI) model of diffuse mTBI, we evaluated the extent of acute neuroinflammation and its relation to axons undergoing DAI in the micro pig 6 h following injury. Large amyloid precursor protein (APP) containing swellings, indicative of the proximal segments of axons undergoing DAI, were found diffusely scattered throughout the brain, with consistent involvement of the thalamus, an area commonly affected in human TBI [49–54]. Acute neuroinflammation accompanied these axonal changes and mapped to the same sites within the thalamus. Importantly, activated microglial processes converged on proximal axonal swellings undergoing DAI to form increased numbers of physical contacts as compared to the number of contacts made between microglia and uninjured axons in sham animals.

## Methods

### Animals

Experiments were conducted in accordance with the Virginia Commonwealth University institutional guidelines concerning the care and use of laboratory animals (Institutional Animal Care and Use Committee), which adhere to regulations including, but not limited to, those set forth in the “Guide for the Care and Use of Laboratory Animals: 8th Edition” (National Research Council). Twenty-one adult male Yukatan micro pigs, weighing 15–25 kg (~6 months of age), were used for this study. Animals were housed in environmentally controlled pens in pairs on a 12-h light–dark cycle, with free access to food and water.

### Surgical preparation and injury induction

Micro pigs were initially anesthetized with an intramuscular injection of 100 mg/ml xylazine (2.2 mg/kg; AnaSed Injection, Shenandoah, IA, USA) and 100 mg/ml Telazol (2.0 mg/kg; tiletamine HCL and zolazepam HCL; Pfizer, New York, NY, USA) followed by intravenous administration of sodium pentobarbital (60 mg/kg; Sigma-Aldrich, St. Louis, MO, USA). Once the absence of a corneal reflex was verified, the micro pig was intubated and ventilated with 1–2 % isoflurane mixed in 100 % oxygen throughout the experiment. Ophthalmic lubricant (Dechra, Overland Park, KS, USA) was applied to avoid damage or drying of the eye. Body temperature was monitored with a rectal thermometer and maintained at 37 °C with a heating pad. Catheters were placed in the right femoral artery and vein for continuous monitoring of mean arterial blood pressure (MABP), assessment of blood gases, and infusion of Lactated Ringer’s (Hospira, Lake Forest, IL, USA) to maintain hydration. A midline incision was made from the supraorbital process to the nuchal crest and a 14-mm-diameter circular craniotomy was trephined along the sagittal suture, positioning the center of the craniotomy 15 mm anterior to lambda, which is on the nuchal crest, and leaving the dura intact. A stainless steal custom threaded hub (Custom Design and Fabrication, Richmond, VA, USA) was screwed into the craniotomy site to a depth of ~4 mm. Screws were then placed directly posterior and anterior-lateral to the craniotomy, and dental acrylic (methyl-methacrylate; Hygenic Corp., Akron, OH, USA) was applied around the hub and screws to insure hub stability. The procedures used to induce cFPI in the micro pig were consistent with those described previously in the rodent [55]. This injury is induced by releasing a pendulum to impact a fluid-filled cylinder that generates a fluid pulse. The fluid pressure wave is transduced through the injury hub to the surface of the dura and ultimately to the cerebral spinal fluid and brain. Briefly, anesthetized micro pigs were connected to a central fluid percussion device retrofitted with a L-shaped stainless steal adaptor that allowed for a sealed connection to the injury hub. Micro pigs were then injured at a magnitude of 1.68 ± 0.4 atm with a pressure pulse measured by a transducer affixed to the injury device and displayed on an oscilloscope (Tektronix, Beaverton, OR, USA). Immediately after injury induction, animals were disconnected from the injury device, the screws and hub were removed from the bone, and the dental acrylic, hub, and screws were removed en bloc. This injury did not result in any breach of the dura mater. Gel foam was placed over the craniotomy/injury site, to alleviate minute bone bleeding, and the scalp was sutured. Animals were maintained under anesthetic for the duration of the 6-h post-injury monitoring period. Identical surgical procedures were followed for sham-injured animals, without the release of the pendulum to induce the injury.

### Physiological assessment

To preclude the possibility that observed pathology was associated with TBI-induced systemic abnormalities, detailed physiological assessments were performed throughout the 6-h post-injury monitoring period. Heart rate, arterial blood pressure, rectal temperature, and hemoglobin oxygen saturation were monitored and recorded throughout the experiment via a Cardell® MAX-12HD (Sharn Veterinary, Inc., Chicago, IL, USA). The femoral artery was cannulated for continuous monitoring of MABP and for blood sampling to determine arterial oxygen tension (PaO_2_), arterial carbon dioxide pressure (PaCO_2_), and pH values using a Stat Profile pHOx (NOVA Biomedical, Waltham, MA, USA). The resting PaCO_2_ level was maintained between 35 and 40 mmHg by adjusting the rate and/or tidal volume of the respirator. All animals maintained physiological homeostasis (i.e., 60 mmHg < MABP < 90 mmHg, hemoglobin oxygen saturation >90 %, 90 BPM < heart rate < 140 BPM; Table 1).

**Table 1:** Systemic physiology was within normal ranges throughout the 6-h post-injury monitoring period

Variable	Group	Pre-injury	Post-injury
Weight	Sham	19.13 ± 4.72	
	TBI	20.12 ± 3.37	
pH	Sham	7.47 ± 0.03	7.48 ± 0.03
	TBI	7.49 ± 0.03	7.52 ± 0.02*
paCO_2_ mmHg	Sham	39.23 ± 4.20	37.92 ± 1.37
	TBI	40.83 ± 2.81	37.61 ± 1.11
paO_2_ mmHg	Sham	585.25 ± 53.92	359.74 ± 187.80
	TBI	558.50 ± 41.42	492.18 ± 116.09
MABP mmHg	Sham	94.29 ± 14.94	86.13 ± 20.19
	TBI	89.53 ± 8.83	79.90 ± 8.24
Hemoglobin O_2_ (%)	Sham	99.90 ± 1.74 × 10^−14^	99.19 ± 0.61
	TBI	99.83 ± 0.167	99.50 ± 0.58

*TBI* traumatic brain injury, *MABP* mean arterial blood pressure

*Significant difference compared with sham values at same measurement point *p* < 0.05. Values are mean ± standard deviation of the mean

### Tissue processing

At 6-h post-sham or cFPI, micro pigs were overdosed with 3-ml euthasol euthanasia-III solution (Henry Schein, Dublin, OH, USA) transcardially perfused with 0.9 % saline followed by 4 % paraformaldehyde/0.2 % glutaraldehyde in Millonig’s buffer (136 mM sodium phosphate monobasic/109 mM sodium hydroxide) for immunohistochemical analysis. After transcardial perfusion, the brains were removed and post-fixed in 4 % paraformaldehyde/0.2 % glutaraldehyde/Millonig’s buffer for 36–48 h. Post-fixed brains were blocked into 5-mm coronal segments throughout the rostral-caudal extent using a tissue slicer (Zivic Instruments, Pittsburgh, PA, USA). In our hands, cFPI in the micro pig produced symmetrical bilateral microscopic pathology, which involved multiple brain loci. The burden of DAI, however, was particularly consistent within the thalamus, leading us to focus on this region for the current communication. Segments containing the thalamus were bisected at the midline and the left side was analyzed. The 5-mm coronal segments containing the thalamus were coronally sectioned in 0.1 M phosphate buffer with a vibratome (Leica, Bannockburn, IL, USA) at a thickness of 40 μm. Sections were collected serially in six-well plates (240 μm between sections in each well) and stored in Millonig’s buffer at 4 °C. For the quantification of both axonal injury and microglial activation, a random well (1–6) was selected using a random number generator and six sections representing the rostral-caudal axis contained within the selected well were analyzed. All histological analyses were restricted to the thalamus using anatomical landmarks and were performed by an investigator blinded to animal injury (sham or mTBI).

### Detection and assessment of axonal injury

To visualize the breakdown of axonal transport within the axonal segment proximal to its neuronal soma, a process indicative of DAI, immunofluorescence targeting the normally expressed and anterogradely transported amyloid precursor protein (APP) was performed.

For the preliminary assessment of DAI in the micro pig brain following cFPI sections from various brain regions throughout the rostra-caudal extent were blocked and permeabilized in 1.5 % Triton/10 % NGS/PBS followed by overnight incubation with the primary rabbit antibody against the C-terminus of β-APP (1:700; Cat.# 51–2700, Life Technologies, Carlsbad, CA, USA) in 10 % NGS/PBS at 4 °C. A biotinylated goat anti-rabbit IgG (1:1000; Cat.# BA-1000, Vector Laboratories, Burlingame, CA, USA) secondary antibody was used. The sections were then incubated in avidin biotinylated enzyme complex using the Vectastain ABC kit (Vector Laboratories, Burlingame, CA, USA) followed by visualization with 0.05 % diaminobenzidine/0.01 % H_2_O_2_/0.3 % imidazole/PBS. The tissue was mounted, dehydrated, and cover-slipped. Visualization of APP-labeled axonal swellings was performed using a Nikon Eclipse 800 microscope (Nikon, Tokyo, Japan) equipped with an Olympus DP71 camera (Olympus, Center Valley, PA, USA).

To quantify the degree of thalamic DAI, immunofluorescence was used, in order to differentiate between the axonal swellings that contain a large amount of APP and the neuronal soma that contain lower amounts of APP. Briefly, six thalamic sections per animal (as detailed above) were blocked and permeabilized in 10 % normal goat serum and 1.5 % Triton followed by overnight incubation with a primary rabbit antibody against the C-terminus of β-APP (1:700; Cat.# 51–2700, Life Technologies, Carlsbad, CA, USA) at 4 °C. Secondary antibody, Alexa Fluor 568-conjugated goat anti-rabbit IgG (1:500; Cat.# A-11011, Life Technologies, Carlsbad, CA, USA) was then incubated and the tissue was mounted using Vectashield hardset mounting medium with Dapi (Cat.# H-1500; Vector Laboratories, Burlingame, CA, USA). Tissue from all animals was processed concomitantly to obviate variability in staining intensity. Visualization of APP-labeled axonal swellings was performed using a Nikon Eclipse 800 microscope (Nikon, Tokyo, Japan) equipped with an Olympus DP71 camera (Olympus, Center Valley, PA, USA). Image acquisition settings were held constant for all animals. Images (60 images per animal; 10 images in each of the 6 sections assessed) were taken by a blinded investigator at 10× magnification (0.72-mm^2^ field) in a systematically random fashion starting at the dorsal lateral aspect of the thalamus. Dapi signal was used for field advancement and to verify focus as well as restriction within the thalamus. A fluorescent intensity threshold was set for all images to eliminate any neuronal somatic expression of APP from the assessment. The number of APP^+^ axonal swellings was analyzed using the particle analysis function in ImageJ software (NIH, Bethesda, MD, USA). The number of APP^+^ swellings per unit area was quantified for each image and averaged for each animal.

### Detection and semi-quantification of microglia activation

To identify microglia, immunohistochemistry against the calcium binding protein, Iba-1 (1:1000; Cat.# 51–2700, Life Technologies, Carlsbad, CA, USA), was done. Briefly, six sections per animal (as explicated above) were blocked and permeabilized in 1.5 % Triton/10 % NGS/PBS followed by overnight incubation with the primary antibody in 10 % NGS/PBS at 4 °C. A biotinylated goat anti-rabbit IgG (1:1000; Cat.# BA-1000, Vector Laboratories, Burlingame, CA, USA) secondary antibody was used. The sections were then incubated in avidin biotinylated enzyme complex using the Vectastain ABC kit (Vector Laboratories, Burlingame, CA, USA) followed by visualization with 0.05 % diaminobenzidine/ 0.01 % H_2_O_2_/0.3 % imidazole/PBS. The tissue was mounted, dehydrated, and cover-slipped. Tissue from all animals was processed concomitantly to reduce variability between animals. The diffuse nature of the microscopic pathology, as well as the extensive area of the micro pig thalamus, precluded the use of traditional stereological quantification. Therefore, the entire thalamus was assessed for each of the six sections selected for each animal. Visualization of Iba-1-labeled microglia was performed using a Nikon Eclipse 800 microscope (Nikon, Tokyo, Japan). Identification of microglia activation was based on specific morphological criteria. Microglia with highly ramified fine process networks that were lightly labeled with Iba-1 were considered non-reactive, while microglia with thicker, shorter, or absent processes and darker Iba-1 labeling were identified as active/reactive [25–27]. The degree of microglia activation was assessed using a graded scale from 0 to 5 (0 = no microglial activation observed, 1 = ramified microglia with thicker processes and darker Iba-1 labeling observed in ~5 % of the thalamus, 2 = activated microglia observed in ~5–10 % of the thalamus, 3 = activated microglia observed in ~10 < 25 % of the thalamus, 4 = activated microglia observed in ~25 < 50 % of the thalamus, and 5 = activated microglia observed in >50 % of the thalamus). Two blinded investigators analyzed all sections independently and their scores were averaged for each animal.

### Tissue processing for microglia morphology and process contact analyses

A subset of tissue sections taken from 9 injured and 3 sham animals were triple labeled with the following antibodies: rabbit anti β-APP (1:700; Cat.# 51–2700, Life Technologies, Carlsbad, CA, USA), rabbit anti Iba-1 (1:1000; Cat.# 019-19741, Wako, Osaka, Japan), and rat anti-myelin basic protein (1:1000; Cat#NB600-717, Novus Biologicals, Littleton, CO, USA). To reduce the amount of lipid within the tissue and enhance antibody penetration, the sections were dehydrated then rehydrated through varying percentages of ethanol, with the tissue beginning and ending in PBS. The tissue was blocked and permeabilized with 10 % NGS/1.5 % Triton/PBS followed by incubation with the rabbit anti-APP antibody over night at 4 °C followed by incubation with Alexa Fluor 488-conjugated goat anti-rabbit secondary antibody (1:500; Cat.# A11034, Life Technologies, Carlsbad, CA, USA). Tyramide amplification of the APP signal (primary antibody diluted to 1:5000) was performed on the majority of sections to further reduce the risk of cross-reaction with the Iba-1 antibody, using a rabbit Alexa-488 conjugated Tyramide amplification kit (Cat.# T20922, Life Technologies, Carlsbad, CA, USA) according to the manufacturer’s instructions. Following APP immunolabeling, the tissue was blocked once again with 10 % NGS then incubated with the rabbit anti-Iba-1 and rat anti-MBP antibodies. Alexa Fluor 568-conjugated goat anti-rat IgG (1:500; Cat.# A11077, Life Technologies, Carlsbad, CA, USA) and Alexa Fluor 633-conjugated goat anti-rabbit IgG (1:500; Cat.# A21071, Life Technologies, Carlsbad, CA, USA) were used for the visualization of MBP and Iba-1, respectively. Controls to assess cross-reactivity among all antibodies were performed using the following antibody combinations: no primary/rabbit Alexa-488 (or rabbit Alexa-488 tyramide amplification), rat Alexa-568, rabbit Alexa-633; rat anti-MBP primary/rabbit Alexa-633 secondary; rabbit anti-Iba-1 primary/rat Alexa-568 secondary; rabbit anti-APP primary (either 1:700 or 1:5000 followed by second blocking step/rabbit Alexa-633 secondary). For all controls, any cross-reactivity was below background detection limits (data not shown). Tissue was mounted using Vectashield hardset mounting medium with Dapi (Cat.# H-1500; Vector Laboratories, Burlingame, CA, USA) and *z*-stacked images (10–25-μm-thick stacks; 0.32 μm between steps; sham = 6 *z*-stacks, injured = 19 *z*-stacks) were captured on a Zeiss LSM 710 confocal microscope (Carol Zeiss, Oberkochen, Germany). Regions for imaging were selected based on the APP profile for injured animals and the penetration of MBP for the sham animals. Three-dimensional reconstructions of the *z*-stacks were made using Velocity software (PerkinElmer, Waltham, MA, USA).

### Microglia morphology analysis

For the morphological analysis of microglia, 2D representations of the Iba-1-labeled Alexa 633 channel 3D reconstructions (sham = 1 reconstruction from each of 3 animals; injured = 1 reconstruction from each of 9 animals) were used. Each individual cell with an identifiable soma and process network within the reconstruction was assessed. The soma size, the total number of processes (with each new branch point designating the origin of a new process), the average process length, the number of primary processes that directly originate from the soma, and the number of terminal process endpoints/tips for each microglial cell were quantified manually using ImageJ software (NIH, Bethesda, MD, USA). These morphological features and particularly the assessment of total number of processes and the number of terminal process endpoints provide detailed data indicating the complexity/ramification of the process network of individual microglia.

### Microglia process contact analyses

Triple-labeled 3D reconstructions performed using Velocity software were used for the analysis of microglia process contacts with DAI in injured micro pigs or intact axonal segments in sham animals. All APP+ axonal swellings contained within each 3D image were assessed in injured animals and 10 myelinated fibers per 3D image, chosen via random-number generator determined *x*, *y* coordinates, were assessed for sham animals. The length of the proximal axonal swelling (from APP+ axonal stem to the disconnected base of APP+ swelling) or the intact myelinated MBP+ axonal segment for sham animals was measured using Velocity software. Microglia process contacts were identified manually on 3D images and confirmed by stepping through the *z*-stacks. All microglial contacts within the delineated axonal segments were included in the analysis.

### Electron microscopy

To evaluate the ultrastructural characteristics of DAI in the micro pig thalamus while verifying microglia process contacts on these axonal swellings, a subset of tissue was immunolabeled with either rabbit anti β-APP (1:700; Cat.# 51–2700, Life Technologies, Carlsbad, CA, USA) or rabbit anti Iba-1 (1:1000; Cat.# 019–19741, Wako, Osaka, Japan), followed by incubation with biotinylated goat anti-rabbit IgG (1:1000; Cat.# BA-1000, Vector Laboratories, Burlingame, CA, USA) secondary antibody. The reaction product was visualized with 0.05 % diaminobenzidine/0.01 % hydrogen peroxide/0.3 % imidazole in 0.1 M phosphate buffer and the tissue was prepared for EM analysis. In this approach, tissue sections were osmicated, dehydrated, and embedded in epoxy resin on plastic slides. After resin curing, the slides were studied with routine light microscopy to identify the precise thalamic areas for excision. Once identified, these sites were removed, mounted on plastic studs, and 70-nm sections were cut serially and mounted on Formvar-coated slotted grids. The grids were stained in 5 % uranyl acetate in 50 % methanol and 0.5 % lead citrate. Ultrastructural qualitative analysis was performed using a JEOL JEM 1230 transmission electron microscope (JEOL-USA, Peabody, MA, USA) equipped with Ultrascan 4000SP CCD and Orius SC1000 CCD cameras (Gatan, Pleasanton, CA, USA).

### Statistical analysis

Data was tested for normality using a Shapiro-Wilk analysis. Normally distributed data was analyzed via one-way analysis of variance (ANOVA). Non-parametric data were analyzed using a Mann–Whitney *U* test. Statistical significance was set at a *p* value <0.05. Data are presented as mean ± standard error of the mean (SEM).

## Results

### Mild diffuse traumatic brain injury does not generate physiologic or macroscopic pathology in the pig

**Fig. 1:**
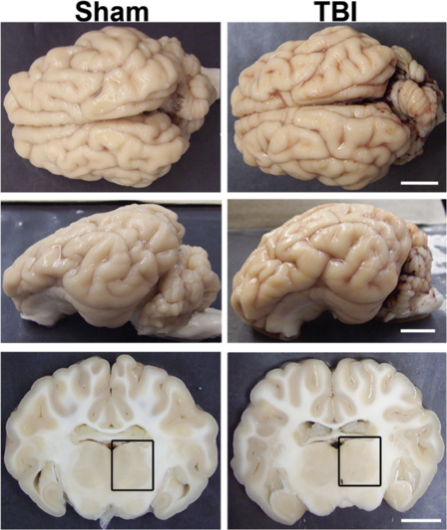
No macroscopic pathology is observed in the micro pig brain following mild traumatic brain injury. Representative photographs of the gross micro pig brain 6 h following sham or cFPI. The *top panel* is a dorsal view while the *middle panel* is a lateral view of the whole pig brain. The *lower panel* represents 5-mm-thick coronal sections taken at approximately the level of bregma. The *boxes* indicate the regions of analysis of both axonal injury and microglia activation in the thalamus. Note that the cFPI model employed did not result in contusion or hematoma formation and only minimal subarachnoid hemorrhage was apparent, consistent with mild diffuse injury. Scale bar: 10 mm

The cFPI used in this study has been successfully employed in rodents for decades to mimic the movement of the brain within the cranial vault following non-contusive diffuse mTBI with high efficacy and consistency [55]. This injury generated virtually no macroscopic pathology in the micro pig brain (Fig. 1). While limited subarachnoid bleeding, particularly overlying the occipital cortex and cerebellum, was observed, macroscopic hemorrhage within the brain parenchyma was not detected. Isolated petechial hemorrhage was observed in a few injured animals, however, this did not interfere with any of the analyses performed. Additionally, these injuries were not accompanied by contusion, hematoma formation, ventricular enlargement, or tissue loss throughout the rostral-caudal extent of the micro pig brain. Collectively, these features speak to the mild diffuse nature of the injury employed.

To evaluate the possibility of confounding systemically induced change in this model, each animal’s systemic physiology was closely monitored for the duration of the experiment, both prior to the induction of injury and for the entire 6-h post-sham or injury period. Core temperature was also monitored and maintained at 37 °C to negate the possibility of protection due to hypothermia. As depicted in Table 1, averages of the pre- and post-injury physiologic values, taken throughout the 6-h post-injury monitoring period, were primarily consistent with sham-injured control animals (one-way ANOVA, weight *F*_1,19_ = 0.199, *p* = 0.661; pre-injury pH *F*_1,19_ = 2.169, *p* = 0.157; pre-injury paCO_2_*F*_1,19_ = 0.732, *p* = 0.403; post-injury paCO_2_*F*_1,19_ = 0.199, *p* = 0.660; pre-injury paO_2_*F*_1,19_ = 1.000, *p* = 0.330; post-injury paO_2_*F*_1,19_ = 2.860, *p* = 0.107; pre-MABP *F*_1,15_ = 0.150, *p* = 0.704; post-injury MABP *F*_1,15_ = 0.115, *p* = 0.739; pre-injury hemoglobin O_2_*F*_1,19_ = 0.435, *p* = 0.518; post-injury hemoglobin O_2_*F*_1,19_ = 0.739, *p* = 0.401; sham *n* = 3, mTBI *n* = 18). Post-injury pH, however, was slightly higher than sham (*F*_1,19_ = 6.174, *p* = 0.022). Importantly, these values were all within normal ranges throughout the experiment (Table 1). The paO_2_, both prior to and following injury, was higher than typically reported due to our use of 100 % O_2_. While these values are higher than are typically observed, the paO_2_ remained well below the lower limit for oxygen toxicity [56, 57]. Importantly, since all physiologic parameters evaluated were within normal ranges, pathology observed could be attributed to the mTBI and not to additional systemic physiological changes.

### Extensive DAI is apparent in the thalamus following mTBI

**Fig. 2:**
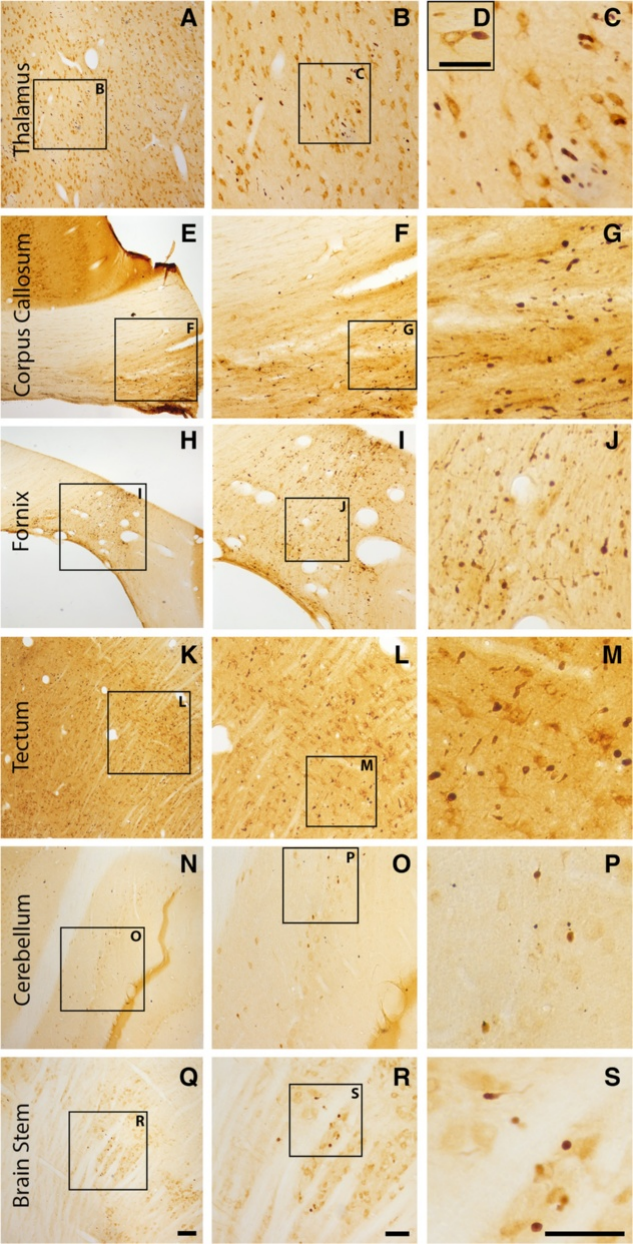
Axonal injury is observed in various regions throughout the micro pig brain following cFPI. Representative photomicrographs of APP immunohistochemistry in regions of the micro pig brain that demonstrated DAI in animals sustaining cFPI. Images in the *middle panel* (**b**, **f**, **i**, **l**, **o**, **r**) are magnified regions indicated in the images of the *left panel* (**a**, **e**, **h**, **k**, **n**, **q**) and images in the *right panel* (**c**, **g**, **j**, **m**, **p**, **s**) are magnified regions indicated in the *middle panel* (**b**, **f**, **i**, **l**, **o**, **r**), respectively. Note that DAI within the thalamus and tectum was diffusely distributed throughout the domain, while DAI within the other regions was more localized. Also note that while not common, APP+ proximal axonal swellings in continuity with the neuronal soma (**d**) were observed in the thalamus. Scale bar in **q**: 200 μm; **r** and **s**: 100 μm; **d**: 50 μm

**Fig. 3:**
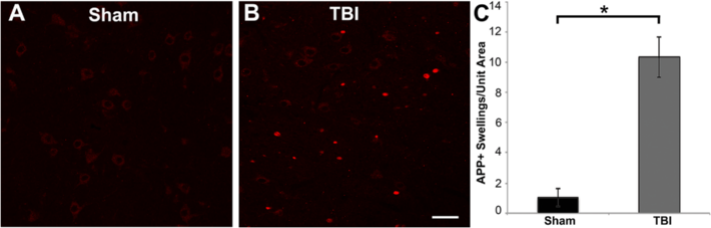
Abundant DAI is readily apparent 6 h following cFPI in the micro pig thalamus. Representative photomicrographs of APP immunofluorescence in the thalamus of animals sustaining sham (**a**) or cFPI (**b**). While sham-injured animals had little to no APP labeling, prevalent APP+ axonal swellings, indicative of DAI, were apparent following injury. **c** Bar graph depicting the average number of APP labeled axonal swellings/ 0.72 mm^2^ of thalamic tissue. Graph depicts mean ± standard error of the mean. *p < 0.05. Scale bar: 50 μm

Since axonal injury is a pathological hallmark associated with much of the morbidity following TBI [10, 11, 14–18], the extent of DAI was qualitatively assessed in various regions of the micro pig brain 6 h following sham or cFPI. This time point was chosen, based on our initial observations in this model, which revealed robust DAI by 6-h post-injury in the micro pig brain (unpublished findings). Pronounced DAI, identified as APP+ proximal axonal swellings, indicative of impaired protein transport in the proximal axonal segment remaining attached to the neuronal soma following disconnection [53, 54, 58, 59], was observed in the thalamus, corpus callosum, fornix, tectum of the midbrain, cerebellum, and brainstem (Fig. 2). While, in some animals, other brain regions displayed more densely localized DAI, diffuse thalamic DAI was the most consistent finding across animals. As thalamic damage is also a common occurrence in human TBI [49–52], we concentrated our quantitative histological analysis on this anatomical region (one-way ANOVA *F*_1,19_ = 6.677, *p* = 0.018; sham *n* = 3, TBI *n* = 18; Fig. 3). Consistent with previous studies examining DAI via APP accumulation, no APP+ axonal swellings were observed in sham-injured micro pigs (Fig. 3a). Following cFPI, however, substantial APP+ proximal axonal swellings were apparent throughout the thalamic domain (Fig. 3). DAI within the micro pig thalamus appeared as large (~5 μm in diameter) APP+ spheroids diffusely distributed in patches throughout the dorsal-ventral and rostral-caudal extent of the thalamus.

**Fig. 4:**
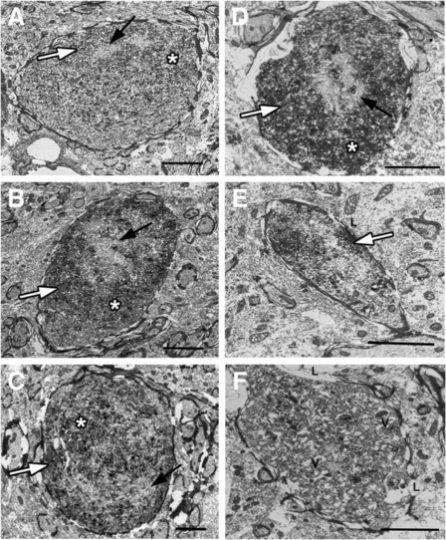
Ultrastructural characteristics of acute DAI within the micro pig thalamus are consistent with human DAI. Representative electron micrographs of axonal swellings labeled with (**a**–**e**) or without (**f**) APP (*white arrows*). **a**–**e** Consistent with ultrastructural axonal pathology in humans, APP+ proximal axonal swellings in the pig following central fluid percussion injury display clumped disordered neurofilaments (*black arrows*) and areas of organelle accumulation (*asterisks*). **f** Distal axonal swellings, lacking APP labeling, demonstrate characteristics consistent with Wallerian degeneration, including, vacuolization (V) and lucent zones surrounding aspects of the swelling indicating axolemmal or myelin disruption (L). Scale bar **b**–**e**: 2 μm

To explore the subcellular pathology of DAI in the micro pig thalamus 6 h following diffuse mTBI, the ultrastructure of these APP+ proximal axonal swellings was assessed (Fig. 4). The majority of proximal axonal swellings, as identified by immunoelectron microscopy against APP, demonstrated ultrastructural alterations consistent with axonal damage. Disordered and clumped neurofilaments were commonly observed within APP containing axonal swellings, with some cases displaying a neurofilamentous core surrounded by organelles (Fig. 4c). Other proximal swellings exhibited predominant organelle accumulation. These findings were consistent with ultrastructural descriptions of human TBI-induced acute axonal pathology [60]. These subcellular changes in the proximal axonal swellings were not, however, consistent with the initiation of Wallerian degeneration [12, 58], which was observed in distal axonal swellings that lacked APP immunoreactivity (Fig. 4f). These APP− distal axonal swellings displayed vacuolization and/or increase cytoplasmic electron density consistent with Wallerian change. Distal, APP−, axonal swellings also displayed areas of axolemmal or myelin disruption, reflected in lucent zones surrounding aspects of the swelling (Fig. 4f).

### Acute thalamic microglia activation following mTBI occurs in areas sustaining DAI

**Fig. 5:**
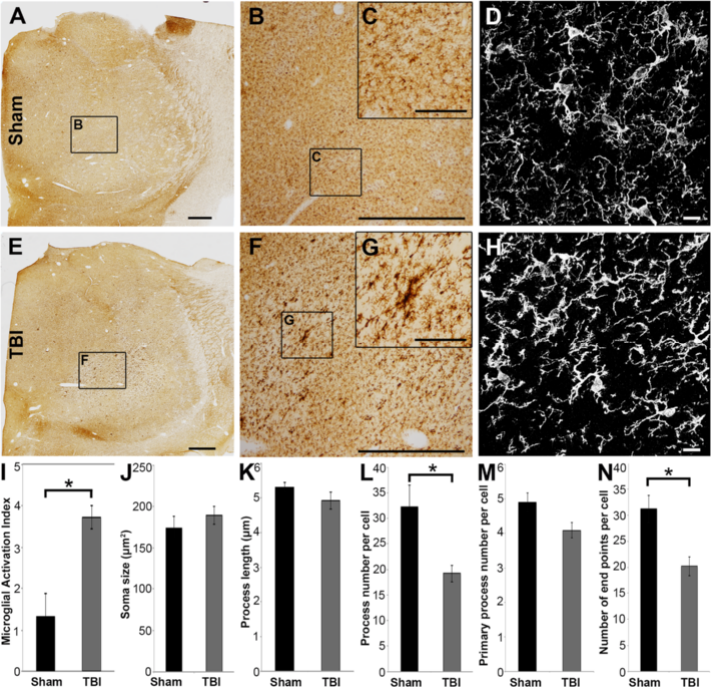
Extensive microglial activation is observed in the micro pig thalamus 6 h following diffuse mTBI. Representative photomicrographs of the microglial marker Iba-1 in the thalamus of sham-injured (**a**–**d**) or central fluid percussion injured (**e**–**h**) micro pigs. **b** and **f** are magnified regions indicated in **a** and **e**, and **c** and **d** are magnified regions indicated in **b** and **f**, respectively. **d** and **h** are two-dimensional flattened images of three-dimensional stacks through microglia in the sham (**d**) or injured thalamus (**h**). Note that the microglia appear ramified in sham-injured animals, indicating a quiescent state. While ramified microglia are present in brain-injured pigs, a large proportion of microglia have retracted, amoeboid or stellate morphologies, indicating activation. Bar graphs illustrating the degree of microglial activation in the thalamus (**i**) as well as the average soma size (**j**), average process length (**k**), average process number (**l**), average number of primary processes (**m**), and average number of end points (**n**) per Iba-1+ cell. Scale bar **a** and **e** = 1 mm, **b** and **f** = 200 μm, **c** and **g** = 40 μm, **d** and **h** = 10 μm. Graph depicts the mean ± standard error of the mean. *p < 0.05

Neuroinflammation, as identified by Iba-1+ microglia with activated morphologies, within the micro pig thalamus was assessed using a graded scale from 0 to 5 (0 = no observed microglial activation and 5 = activated microglia observed in >50 % of the coronal thalamic section; sham *n* = 3, TBI *n* = 18). While the microglia within the sham thalami were evenly distributed and primarily non-reactive, with spindly ramified process networks that were lightly labeled with Iba-1, some isolated microglia demonstrated thicker, shorter processes with more substantial Iba-1 labeling (Fig. 5a–d). Following cFPI, however, microglia activation was pervasive, with pockets of morphologically active microglia, exhibiting heavy Iba-1 labeling, thicker processes, and less complex process networks, dispersed throughout the micro pig thalamus (Mann–Whitney *U* test, *p* = 0.006; Fig. 5e–h).

The process network of individual microglial cells was analyzed in either sham or injured thalami to verify the morphological alterations observed using the above semi-quantitative assessment (Mann–Whitney *U* test: soma size *p* = 0.613, process length *p* = 0.994, total process number *p* = 0.001, primary process number *p* = 0.059, process endpoints *p* = 0.001; sham *n* = 30 cells, TBI *n* = 70 cells). The microglia in sham-injured micro pigs had ramified/complex process networks, reflected primarily by the total process number and the number of terminal process endpoints per cell (Fig. 5). Microglia within the thalamus of micro pigs sustaining mTBI-induced DAI, however, had fewer overall processes and less terminal process endpoints, indicative of a less complex and/or ramified process network (Fig. 5i, l, n). While the soma size, number of primary processes, and the average process length remained comparable between sham microglia and microglia following mTBI, the drastic reduction in the complexity/ramification of the microglial process network with injury confirms morphological alterations consistent with resident microglial activation [25–27, 61, 62] .

**Fig. 6:**
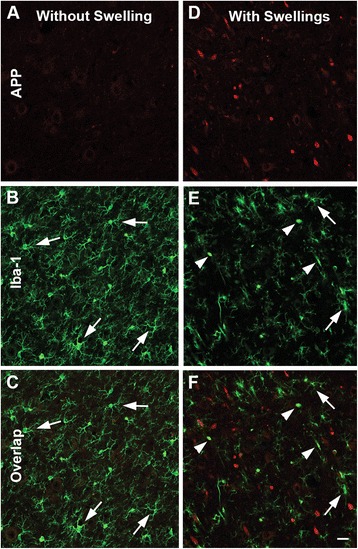
Microglia activation occurs in thalamic sectors sustaining acute DAI 6 h following mTBI. Representative confocal micrographs of APP (*red*; **a** and **d**) and Iba-1 (*green*; **b** and **e**), with overlays in **c** and **f**, in the thalamus of the same injured animal. Interestingly, areas lacking axonal injury (**a**–**c**) appear to contain inactive ramified microglia (*arrows*), whereas thalamic sites exhibiting DAI, indicated by accumulation of APP in axonal swellings (**d**–**f**), also appear to contain the majority of morphologically activated Iba-1+ microglia (*arrow heads*). Scale bar: 20 μm

Interestingly, the degree of microglial activation within the thalamus, as assessed via the microglial activation score, was significantly correlated to the amount of DAI observed in each animal (Spearman’s rho correlation coefficient = 0.763, *p* = 0.000; *n* = 21). Additionally, the areas of dense microglial activation appeared consistent with the thalamic sites that demonstrated the highest degree of DAI. Therefore, the spatial relationship between activated Iba-1+ microglia and DAI within the injured thalamus was assessed. As depicted in Fig. 6, regions that contained DAI also contained a plethora of morphologically activated microglia, whereas, areas in the injured thalamus that did not sustain DAI primarily contained non-reactive, ramified microglia.

### Microglia processes preferentially contact DAI proximal axonal swellings acutely post-injury

**Fig. 7:**
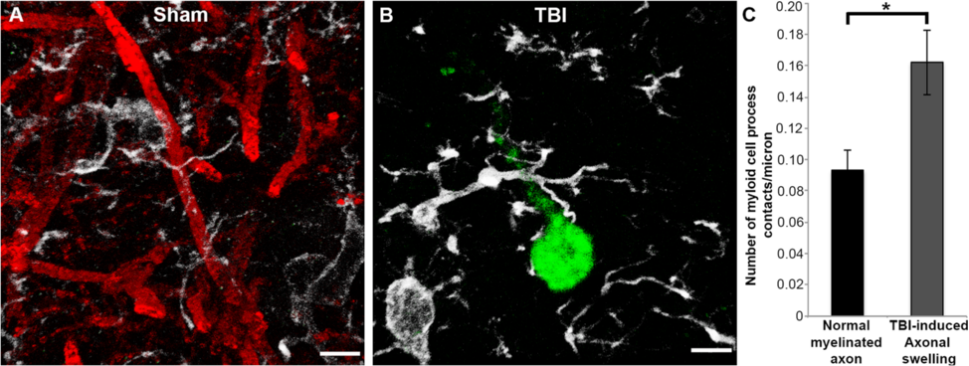
Microglia processes appear to preferentially contact TBI-induced proximal axonal swellings. Representative 3D reconstructions of MBP+ myelinated axons (*red*) or APP+ axonal swellings (*green*) and Iba-1+ microglia (*white*) in sham-injured (**a**) or central fluid percussion injured (**b**) thalami. **c** Bar graph depicting the average number of Iba-1+ microglial processes contacting either MBP+ myelinated fibers in the sham animals or APP+ axonal swellings in injured animals. Graph depicts the mean ± standard error of the mean. *p < 0.05. Scale bar: 5 μm

To investigate the possibility that activated microglial processes converge on proximal axonal swellings sustaining mTBI-induced DAI in the micro pig thalamus, the number of microglia processes that contacted APP+ axonal swellings in injured animals or normal myelinated axons in sham animals was assessed (Mann–Whitney *U* test *p* = 0.018; sham *n* = 60 axonal segments, TBI *n* = 60 axonal swellings). Following sham injury, microglia processes made contact with myelinated axons, however, these contacts were sparse (Fig. 7a) and primarily consisted of microglia processes passing by and/or crossing over myelinated axons (sham = 63.39 % of total contacts, TBI = 25.23 % of total contacts). This is shown in more detail in an additional movie file (Additional file 1). Conversely, nearly double the number of microglia processes contacted APP+ proximal axonal swellings following diffuse mTBI (Fig. 7). The majority of these contacts were bulbous end processes (TBI = 58.16 % of total contacts, sham = 36.61 % of total contacts) as apposed to processes passing over axons, which were common in the sham. An additional movie file represents these finding in more detail (Additional file 2). The percentage of microglial processes that appeared to cradle or “cup” the axon were also increased when contacting APP+ swellings compared to sham myelinated axons (TBI = 16.60 % of total contacts, sham = 6.09 % of total contacts).

**Fig. 8:**
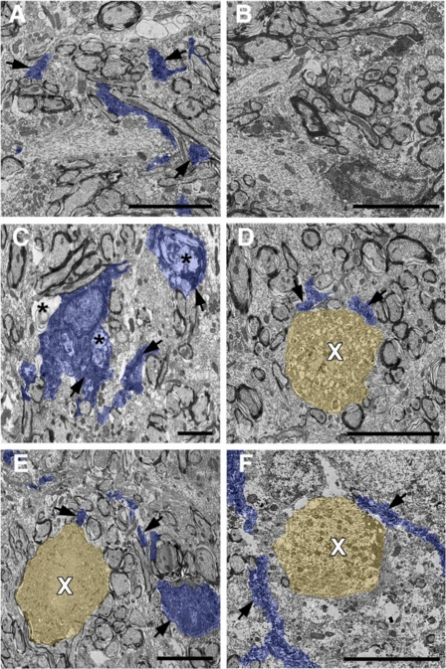
Ultrastructural evidence of non-phagocytic microglial processes directly contacting proximal axonal swellings undergoing acute DAI. Representative electron micrographs of Iba-1 immuno-electron-microscope-labeled microglia in the thalamus of sham (**a** and **b**) and injured (**c**–**f**) animals. Iba-1-labeled structures in **a** and **c**–**f** are *pseudo-colored blue* for clarity. Note that, microglia processes containing Iba-1 (*arrows*) within the thalamus of sham-injured animals (**a** and **b**) are widely dispersed with no apparent association with axonal segments. Following cFPI, however, Iba-1+ microglial processes are much more prevalent. Some phagocytic microglia (**c**), containing material consistent with Wallerian degeneration (*asterisks*), are present following injury. These phagocytic microglia are primarily localized to areas of axonal damage/Wallerian degeneration. **d**–**f** Microglial processes (*pseudo*-*colored blue*) associating with axonal swellings (*pseudo*-*colored yellow*), as identified using the common ultrastructural features observed previously for diffuse axonal injury, appeared most frequently as **d** Iba-1 immunolabeled (*arrows*) puncta adjacent to the axonal swelling (*white X*), however, some cases **e** and **f** allowed for the tracing of microglia processes over short distances. Importantly, the Iba-1+ microglia processes found associated with axonal swellings did not reveal ultrastructural changes consistent with phagocytosis. Scale bar: 5 μm

To further evaluate the increase in microglial process convergence on proximal segments of axons sustaining DAI, qualitative ultrastructural analysis of microglia morphology and process contacts were performed utilizing immunoelectron microscopy targeting Iba-1 (Fig. 8). Microglial cell bodies were easily recognized in sham thalami, however microglial processes were fine and widely dispersed with few processes directly associating with axons (Fig. 8a, b). Following diffuse mTBI, both the Iba-1+ microglial processes and soma were more prominent. As previously described, microglia with myelin and other cellular debris, indicative of active phagocytosis, were present in areas of Wallerian degeneration (Fig. 8c). In the presence of proximal axonal swellings, as ultrastructurally identified using the common features observed and displayed in Fig. 4, however, microglia did not display sub-cellular alterations consistent with phagocytosis. Rather, small Iba-1+ points of contact were observed (Fig. 8d, e). In some cases, the microglial processes could be traced from the site of axonal contact back to the microglia soma, however, the majority of processes passed out of the plane of section (Fig. 8e, f). These data suggest that while microglia do phagocytize debris from the degenerating distal axon, they do not participate in phagocytosis of the proximal axonal swelling acutely post-mTBI, rather microglial processes appear to preferentially contact and/or converge on proximal axonal swellings.

## Discussion

The current study demonstrates acute microglial process convergence on proximal swellings of axons sustaining acute DAI in the micro pig thalamus, an association not previously recognized in the literature. Throughout this study, we employed a cFPI model in adult micro pigs to evaluate the diffuse pathology associated with mTBI in a higher order gyrencephalic animal more comparable to humans [47, 48]. This cFPI model, which has been successfully employed in rodents for decades, involves the transmission of a fluid pressure pulse to the brain and CSF through the intact dura mater, mimicking the movement of the brain following non-contusive diffuse mTBI in a reproducible fashion [55, 58, 63]. Using this model of cFPI, we demonstrated that diffuse mTBI in the micro pig produced substantial DAI, particularly within the thalamus, an area commonly affected in human TBI [49–52], without concomitant contusion or hematoma formation. Importantly, micro pig DAI was shown to be ultrastructurally consistent with that seen in humans [60] indicating that this model replicates human-like features of mTBI-associated diffuse pathology.

In concert with this axonal damage, extensive neuroinflammation, as identified by morphological alterations, indicative of microglial activation [25–27], was observed in the thalamus of micro pigs acutely following mTBI. Neuroinflammation is common following CNS injury in a variety of disease states and has been observed years following TBI in the human population [37, 51, 62, 64–69]. This chronic neuroinflammatory response has recently been observed in rodent models of trauma as well and is thought to be associated with negative outcomes both clinically and experimentally [19–23]. For a decade, it has been accepted that microglia react to CNS injury within minutes [70], however, little is known regarding acute neuroinflammation occurring minutes to hours following diffuse mTBI.

Previous studies, including our own, have focused on the phagocytic activity of microglia sub-acutely (days) following injury in rodents [29, 35–37, 39]. Specifically, we and others have identified an association between microglia phagocytosis and injured axons undergoing Wallerian degeneration days following injury in the rodent. Sub-acute phagocytosis has also been observed following demyelination and axonal injury in models of multiple sclerosis and leukodystrophy as well as during synaptic pruning during normal development [30, 62, 65, 71, 72]. It is possible, due to the fact that the current study only assess microglia at one acute time-point post-mTBI, that the observed process convergence on proximal axonal swellings is an early indication of microglial progression to phagocytic reactivity. Microglia, however, have non-phagocytic functions that are less well understood [28–33]. In healthy brain, endogenous microglia have been shown to survey the parenchyma by extending highly dynamic processes that interact with the other cells of the CNS [73, 74]. This dynamic microglial process remodeling is altered under the influence of neuronal signals [27, 70, 75–77]. Additionally, a low calcium environment can activate microglial process convergence on neurons [78]. Microglial processes have also recently been shown to preferentially contact and functionally alter neurons with specific electrophysiological characteristics [61, 79].

While our study did not explore dynamic process remodeling, rather it assessed a snapshot of process dynamics acutely following mTBI, our findings indicate that non-phagocytic microglia processes preferentially interact with and/or converge on the proximal aspects of diffusely injured axons. Our previous studies and clinical observation indicate that the proximal axonal segments of neurons sustaining DAI do not immediately progress to cell death. Rather, they plastically adapt and remodel [14, 80–82], precluding the likelihood of acute phagocytosis. Calcium influx, however, has been well documented to occur acutely in axons sustaining DAI [83, 84]. This influx might alter the calcium concentration in the extracellular space immediately surrounding the proximal axonal swelling, which could act as a signal for microglial process convergence. Additionally, diffusely injured axons display acute electrophysiological alterations [85–87] which could modulate microglial responses.

Activated microglia are typically categorized into different subgroups of M1, inflammatory microglia, or alternatively activated/anti-inflammatory M2 microglia. These sub-groups, which have been observed in rodents, humans, and pigs, express either pro- (M1) or anti- (M2) inflammatory cytokines and are activated via different signaling pathways [29, 47, 88–91]. The specific functions of M2-activated microglia following mTBI are not well understood, however, it is speculated that M2 microglia are neuroprotective [22, 92, 93]. It is possible that the M2-activated microglia population is preferentially associated with the proximal swellings undergoing DAI, however, due to the limited study of neuroinflammation in pig, further investigation would be required to verify this potential phenomenon.

## Conclusions

In summary, we demonstrated that central fluid percussion injury in adult micro pigs precipitated substantial acute axonal injury in the thalamus that is ultrastructurally similar to acute DAI in humans. Extensive microglial activation was also observed in the same thalamic sites, which directly correlated to the burden of thalamic axonal injury. The physical relationship between activated microglial processes and the proximal swelling of axons sustaining acute DAI was dramatically increased compared to uninjured myelinated thalamic axons in sham animals. In aggregate, these studies reveal acute microglial process convergence on proximal axonal swellings undergoing DAI, an interaction not previously recognized in the literature.

Current clinical techniques have emerged that allow for the visualization of neuroinflammation in the human population via PET imaging [51, 64, 94]. Our current finding that early microglial activation is directly associated with acute DAI in the micro pig, paired with the similarities between pig and human inflammatory profiles and responses [47, 48], suggests that this association could be exploited in humans early in the post-traumatic period. Recent observations indicating similar associations between DAI and neuroinflammation in humans weeks to years following injury lend credence to this possibility [69]. Techniques utilizing PET imaging to visualize human neuroinflammation could be employed to assess areas sustaining acute DAI in TBI patients in a way never before possible. Additionally, alterations in neuroinflammatory biomarkers within the serum following TBI could also be used in conjunction with these imaging techniques to estimate the total burden of diffuse injury and better tailor treatment in the human population. Future studies exploring the temporal relationship between neuroinflammation and the pathological progression of DAI through to sub-acute and even chronic time points in this higher order micro pig model could prove invaluable in understanding the interplay between DAI and neuroinflammation in the gyrencephalic brain.

## Additional files

Additional file 1:
**Processes of ramified microglia primarily pass over normal myelinated fibers in the sham thalamus.** Movie representing 3D reconstructions of confocal *z*-stacks of thalamic sites from sham-injured animals, in which Dapi-labeled nuclei are in *blue*, MBP+ myelinated axons are in *red*, and Iba-1+ microglia are in *white. Purple circles* highlight contacts between microglial processes and normal myelinated fibers. Notice that while only a few microglial processes come in direct apposition with normal myelinated fibers, the majority of microglial processes that do contact myelinated fibers do not terminate at the axon; rather pass over the myelinated fiber. Scale bar: 10 μm. (MP4 16442 kb) Additional file 2:
**Processes of activated microglia terminate on proximal swellings of axons sustaining acute DAI.** Movie representing 3D reconstructions of confocal *z*-stacks of thalamic sites from cFPI animals, in which Dapi-labeled nuclei are in *blue*, MBP+ myelinated axons are in *red*, APP+ proximal axonal swellings are in *green*, and Iba-1+ microglia are in *white. Purple circles* highlight contacts between microglial processes and APP+ proximal axonal swellings. Note that the majority of microglial processes that come into contact with APP+ axonal swellings terminate on the proximal swelling, indicating process convergence. Scale bar: 10 μm. (MP4 12603 kb)

## Abbreviations

ANOVA: analysis of variance
APP: amyloid precursor protein
cFPI: central fluid percussion injury
DAI: diffuse axonal injury
MABP: mean arterial blood pressure
mTBI: mild traumatic brain injury
PaO_2_: arterial oxygen tension
PaCO_2_: arterial carbon dioxide pressure
